# Health Impact of Suspected Interpersonal Violence Against Adults: Retrospective Cohort Study

**DOI:** 10.2196/65191

**Published:** 2025-10-30

**Authors:** Rui Barbosa, Rita Lopes, Tiago Taveira-Gomes, Carla Ponte, Teresa Magalhães

**Affiliations:** 1 Department of Public Health and Forensic Sciences, and Medical Education Faculty of Medicine University of Porto Porto Portugal; 2 MTG Research and Development Lab Porto Portugal; 3 Center for Health Technology and Services Research (CINTESIS@RISE) Porto Portugal; 4 Department of Community Medicine, Information and Decision in Health Faculty of Medicine University of Porto Porto Portugal; 5 Faculty of Health Sciences University Fernando Pessoa (FCS-UFP) Porto Portugal; 6 SIGIL Scientific Enterprises Dubai United Arab Emirates; 7 USF Porta do Sol - ULS Matosinhos Senhora da Hora Portugal; 8 TOXRUN–Toxicology Research Unit University Institute of Health Sciences, Advanced Polytechnic and University Cooperative (CESPU) Gandra Portugal

**Keywords:** interpersonal violence, health outcomes, health risk behaviors, mental health, somatic health conditions, health care diagnosis

## Abstract

**Background:**

Interpersonal violence (IV) has an extensive and profound impact on health, representing a public health concern. Different health outcomes have been identified based on the characteristics of the survivor and the abuser, their relationship, the type of violence perpetrated, and the cumulative effect of multiple violent experiences.

**Objective:**

The main objective of this study was to estimate the likelihood of negative health outcomes, such as substance abuse, mental health, and somatic disorders, occurring in adults presenting a clinical suspicion of IV.

**Methods:**

We performed a retrospective, observational cohort study, using secondary data from electronic health records of adult patients of the Local Health Unit of Matosinhos (ULSM). The control cohort included all patients aged between 18 and 59 years followed at ULSM between January 1, 2008, and May 9, 2024, while the violence cohort included patients within the same age range who were suspected survivors of IV within the same time period. Exposure was defined by the presence of one or more of the IV text expressions or codes in the patient’s electronic health record. Data regarding violence suspicion, comorbidities, and health outcomes were obtained by conducting a text search on clinical notes, as well as *ICD-9* (*International Classification of Diseases, Ninth Revision*) and *ICD-10* (*International Statistical Classification of Diseases, Tenth Revision*) and International Classification of Primary Care 2 codes. The follow-up period was 10 years. To estimate the risk of developing health outcomes, we constructed a cohort model using a Cox proportional hazards model adjusted at baseline for age and sex.

**Results:**

The control cohort included 154,145 patients, and the violence cohort included 36,835 patients. Suspected survivors of IV had a higher hazard ratio of developing alcohol abuse (3.05, 95% CI 2.87-3.24), drug abuse (6.03, 95% CI 4.5-8.06), suicidal ideation (5.50, 95% CI 4.78-6.32), major psychiatric disorder (4.46, 95% CI 4.38-4.53), chronic pain (3.70, 95% CI 3.60-3.81), sleep disorders (3.57, 95% CI 3.46-3.68), use of antidepressants (3.57, 95% CI 3.51-3.64), use of anxiolytics (2.98, 95% CI 2.93-3.03), and eating disorder (2.72, 95% CI 2.06-3.59). Regarding somatic health conditions, suspected violence exposure was linked to a higher hazard ratio for metabolic dysfunction–associated steatotic liver disease (6.91, 95% CI 6.37-7.50), chronic immune inflammatory disorder (3.68, 95% CI 3.93-3.44), asthma (2.64, 95% CI 2.53-2.74), chronic kidney disease (2.49, 95% CI 2.39-2.59), hypercholesterolemia (2.16, 95% CI 2.12-2.20), and early heart disease (2.01, 95% CI 1.93-2.10).

**Conclusions:**

Exposure to violence was linked to a higher likelihood of developing adverse events related to substance abuse, mental, and somatic health. Our findings lead to a deeper understanding of the complex burden of violence on health, uncovering new relationships between IV and health outcomes while validating those already explored.

## Introduction

The World Health Organization (WHO) defines violence as “the intentional use of physical force or power, threatened or current, against oneself, another person, or against a group or community, that either results in or has a high likelihood of resulting in injury, death, psychological harm, maldevelopment, or deprivation.” Thus, it includes active and passive behaviors, mostly of a repetitive nature. It is present across every stage of life, regardless of sex, and in different sociocultural contexts and forms [1].

In Portugal, data from the National Institute of Statistics from 2023 shows that 20.1% of people aged between 18 and 74 years have been survivors of physical or sexual violence in adulthood. Women are primarily affected in the context of intimacy, while men are more often survivors of violence from nonpartners [2].

Interpersonal violence (IV) is no longer regarded only as a criminal justice matter. Its impact on health is extensive and profound, and it is recognized as a significant public health problem, which requires the attention of health care providers [1,3]. Overall, IV has short-, medium-, and long-term effects. In fact, they are not limited to immediate physical trauma but include physiological changes, psychological consequences, and coping strategies that may increase the risk of negative health outcomes, years or decades following the primary exposure [4-7]. There are several mechanisms behind these transformations, some happening in the first years of life [5,8-11]: (1) changes in the brain’s white and grey matter, its volume and functional connective; (2) altered neurotransmitter metabolism; (3) changes in the neuroendocrine stress response; (4) chronic inflammation; (5) impaired glucose metabolism; (6) changes in the microbiome in response to stress; and (7) epigenetic mechanisms through DNA methylation. Many of these alterations seem related to the hypothalamic-pituitary-adrenal (HPA) axis, which integrates and coordinates the central and peripheral aspects of the mammalian stress response. Thus, violence exposure acts as a trigger and places survivors at high risk for the development of physical and mental health illnesses, as well as latent and genetically programmed diseases [5,7].

Several studies describe different health outcomes according to the survivor’s and abuser’s personal characteristics and their relationship, as well as the type of violence perpetrated. Intimate partner violence is shown to be related, for example, with mental health disorders (where genetics have a significant preponderance [11]), substance consumption, and somatic diseases, as obesity, metabolic syndrome, type 2 diabetes (T2D; due to increased cortisol levels and suppressed insulin levels [10]), hypertension (as a result of chronic activation of HPA axis, and autonomic nervous system [9]) among others [6,7,9-13]. Bullying and cyberbullying seem to be associated with depression, anxiety, and somatization symptoms [6]. Sexual abuse is found to be mainly related to depression, anxiety, suicide attempts, substance abuse, sleep disorders, gastrointestinal disorders, or nonepileptiform seizures [5,6,14]. Economic abuse is considered associated with increased depression, anxiety, suicidal ideation, and posttraumatic stress disorder [15].

Furthermore, it must be outlined that survivors of IV frequently experience multiple forms of it [6,16]. Experiencing or being exposed to one type of violence increases the risk of becoming a survivor of other types of violence or inflicting harm on others [17,18]. The association of different types of IV or its reiteration may result in different and potentially more severe negative impacts on health consequences [18,19]. This way, to understand the full burden of IV health outcomes, we should look to the cumulative effect of the multiple experiences of violence during the lifespan [18].

Ultimately, there are still associations to be unveiled and better understood that may revolutionize the approach to survivors. The better we understand the possible long-term outcomes of IV, the more capable doctors are to minimize the burden of violence on survivors’ development and well-being.

The main objective of this study was to estimate the likelihood of negative health outcomes, such as substance abuse, mental health, and somatic disorders, occurring in adults presenting a clinical suspicion of IV. The study also aimed to establish the incidence of individuals with suspected exposure to violence followed in the Local Health Unit of Matosinhos (Unidade Local de Saúde de Matosinhos [ULSM]) and to characterize this population both demographically and according to prior clinical history.

## Methods

### Study Design and Setting

A real-world, retrospective, observational, and analytical cohort study was performed using data from electronic health records (EHR) of adult users of ULSM.

ULSM is a health unit that provides primary, secondary, and tertiary health care, being composed of 1 hospital and 14 primary care centers, serving a population of around 176.774 inhabitants.

The study’s inclusion criteria for the violence cohort were: (1) patients aged between 18 and 59 years; (2) sought the ULSM service between January 1, 2008, and May 9, 2024; (3) having text expressions (according to predefined keywords, described in Table 1) related to IV documented in the clinical notes or codes or at least one *ICD-9* (*International Classification of Diseases, Ninth Revision*) or *ICD-10* (*International Statistical Classification of Diseases, Tenth Revision*) or an International Classification of Primary Care 2 (ICPC-2) IV-related code occurring after the age of 18 years (Table 1); and (4) having at least one appointment at ULSM in the 365 days before the index date. This last criterion was established to ensure the capturing of a population regularly followed at ULSM and to exclude sporadic visitors, such as tourists, thus increasing accuracy in the characterization of the local population.

The control cohort consisted of an incident population from ULSM, between 18 and 59 years old, who sought the ULSM service between January 1, 2008, and May 9, 2024, and had at least one appointment at ULSM in the previous 365 days before the index date.

No exclusion criteria were used in this investigation other than not meeting the inclusion criteria. Formal sample size calculations were not conducted, as the sample consisted of all the patients eligible according to the inclusion criteria.

The two study cohorts were not mutually exclusive; therefore, the same patient could enter the two cohorts at different times during the study period.

The index data corresponded to the date when each patient met all the cohort criteria. The patients’ medical history, also described as prior outcomes, was evaluated using all records available in the preindex period. The follow-up period was 10 years (3650 days).

**Table 1:** Codes and text expressions used to define interpersonal violence suspicion in the studya.

Classification	Code
*ICD-9^b^*	995.80 Adult maltreatment, unspecified 995.81 Adult physical abuse 995.82 Adult emotional or psychological abuse 995.83 Adult sexual abuse 995.84 Adult neglect (nutritional) 995.85 Other adult abuse and neglect
*ICD-10^c^*	T74 Adult and child abuse, neglect, and other maltreatment, confirmed T74.01 Adult neglect or abandonment, confirmed T74.11 Adult physical abuse, confirmed T74.21 Adult sexual abuse, confirmed T74.31 Adult psychological abuse, confirmed T74.51 Adult forced sexual exploitation, confirmed T74.61 Adult forced labor exploitation, confirmed T74.91 Unspecified adult maltreatment, confirmed T74.A1 Adult financial abuse, confirmed T76 Adult and child abuse, neglect, and other maltreatment, suspected T76.01 Adult neglect or abandonment, suspected T76.11 Adult physical abuse, suspected T76.21 Adult sexual abuse, suspected T76.31 Adult psychological abuse, suspected T76.51 Adult forced sexual exploitation, suspected T76.61 Adult forced labor exploitation, suspected T76.91 Unspecified adult maltreatment, suspected T76.A1 Adult financial abuse, suspected
ICPC-2^d^	Z12 Relational problem with partner Z13 Partner behavioral problem Z25 Violent act or event
Text expressions	[Aa]bandon [Aa]gred [Aa]gress [Aa]mea[çc]a [Aa]pert[ãa]o [Aa]pert[õo]es [Bb]alead [Bb]at[ei] [Bb]ofet [Cc]hapad [Dd]estru [Dd]ispar [Ee]mpurr [Es]sfaquead [Ee]sgana [Ee]stalad [Ee]strang [Ff]acad [Hh]umilh [Ii]nsult [Mm]urr [Oo]brigada a [Pp]ancad [Pp]aulad [Pp]r[aá]tica[s]* sex [Pp]edrad [Pp]erseg [Pp]ontap [Ss]apatad [Ss]oco [Ss]ufoc

^a^Patients aged between 18 and 59 years followed in the Local Health Unit of Matosinhos should have at least one of the codes or text expressions to qualify for the violence cohort.

^b^ICD-9: International Classification of Diseases, Ninth Revision.

^c^ICD-10: International Statistical Classification of Diseases, Tenth Revision.

^d^ICPC-2: International Classification of Primary Care, 2nd edition.

### Exposure and Outcome Definitions

Exposure was defined by the presence of one or more of the IV text-expressions or *ICD-9*, *ICD-10*, or ICPC-2 codes in the patient’s EHRs described in Table 1.

### Study Variables

The variable categories included in the study were (1) sex, (2) age, (3) health risk behaviors, (4) mental health disorders, and (5) somatic health conditions.

The health risk behaviors category included the following variables: (1) alcohol abuse and (2) drug abuse.

Mental health disorders encompassed (1) major psychiatric disorders, (2) posttraumatic stress disorder, (3) suicidal ideation, (4) chronic pain, (5) headache, (6) sleep disorders, (7) memory disorders, (8) eating disorders, and (9) psychotropic drug use (sedatives, antidepressants, anxiolytics, and antipsychotics).

Within the somatic health conditions category, the included variables were (1) T2D, (2) hypercholesterolemia, (3) metabolic dysfunction–associated steatotic liver disease (MASLD), (4) hypertension, (5) chronic kidney disease, (6) early heart disease, (7) asthma, (8) chronic inflammatory disorder, (9) cancer, and (10) unspecified illness.

All variables described above were used for cohort characterization, using all information available in the preindex period, within study dates.

The events used to estimate hazard ratios (HRs) included all of the health outcomes included in the categories “health risk behaviors,” “mental health disorders,” or “somatic health conditions” occurring up to 3650 days after the index date.

### Statistical Analysis

Analysis was conducted through local execution of analytical programs developed using VERO technology that were compiled for ULSM target data infrastructure built upon Apache Spark Framework version 3.2.1 and source data harmonized according to the OMOP CDM 5.4 standard. The package implements a complete data engineering pipeline to transform the source data into the final dataset, as well as the execution of statistical analysis and the generation of results reports. Continuous variables were described using the average and SD, while categorical variables were expressed by absolute and relative frequencies. Age was analyzed as a continuous and a categorical variable. Patients were stratified into 10-year age categories in order to better characterize the age distribution of the study’s population. To estimate the risk of developing health outcomes, we constructed a cohort model using a Cox proportional hazards model adjusted at baseline for age and sex. HRs were calculated with 95% CIs. Time at risk was calculated from the index date until the development of the outcome of interest. Patients were censored in case of leaving the database. Missing data were not imputed for any variables. Analysis was performed using only the available data.

### Ethical Considerations

The Health Ethics Committee and Data Protection Officer of ULSM authorized the use of the database for this study, under approval codes 14/CES/JAS of 02-11-2022 and 04/CLPSI/2022 of 03-02-2022. Data processing and analysis were performed using analytical programs developed for this purpose that were sent for execution at ULSM servers. Researchers did not have access to patient-level data. Only aggregated data were shared with the researchers. No data extraction took place in the context of this study. Informed consent was waived by the institution, invoking public health and research interests, following the General Data Protection Regulation. No type of compensation was given to participants.

## Results

### Baseline Characteristics of Incident Population

The baseline characteristics of the control and suspected IV cohorts are described in Table 2.

The population on both cohorts was mostly female (83,636/154,145, 54.3% in the control and 23,521/36,835, 63.9% in the suspected IV cohort), average age was 32.7 (SD 23.6) in the control and 42.6 (SD 19.6) in the IV cohort.

Regarding patients’ clinical history, there was a higher prevalence of both drug and alcohol abuse in the suspected IV cohort. The same was true for the major psychiatric disorders, chronic pain, sleep disorders, and the use of psychotropic drugs.

Regarding somatic health conditions, there was a higher prevalence of all studied conditions in the suspected IV cohort when compared to the control, with the biggest differences concerning cancer (1723/154,145, 1.1% in the control; 5704/36,835, 15.5% in the suspected IV cohort), hypertension (7131/154,145, 4.6% vs 13,343/36,835, 36.2%), and hypercholesterolemia (8401/154,145, 5.5% vs 13,819/36,835, 37.5%).

**Table 2:** Baseline characteristics of control and suspected violence cohorts^a^.

						Control group (n=154,145)			Suspected IV^b^ group (n=36,835)
**Sex**, **n (%)**									
	Female				83,636 (54.3)			23,521 (63.9)	
	Male				70,509 (45.7)			13,314 (36.1)	
**Age** **(years)**									
	Mean (SD)				32.7 (23.6)			42.6 (19.6)	
	Range, n (%)								
		10-19		31,275 (20.3)			1746 (4.7)		
		20-29		36,792 (23.9)			5906 (16)		
		30-39		31,892 (20.7)			8334 (22.6)		
		40-49		29,015 (18.8)			9739 (26.4)		
		50-59		25,171 (16.3)			11,110 (30.2)		
**Cohort components**, **n (%)**									
	Violence codes				108 (0.1)			1909 (5.2)	
	Violence registries				4665 (3)			34,985 (95)	
**Prior outcomes**									
	**Health risk behaviors** **, n (%)**								
		Alcohol abuse		180 (0.1)			1001 (2.7)		
		Drug abuse		9 (0.1)			43 (0.1)		
	**Mental health disorders** **,** **n (%)**								
		Major psychiatric disorder		4765 (3.1)			16,378 (44.5)		
		Posttraumatic stress disorder		22 (0.1)			69 (0.2)		
		Suicidal ideation		41 (0.1)			211 (0.6)		
		Chronic pain		493 (0.3)			4178 (11.3)		
		Headache		1640 (1.1)			2793 (7.6)		
		Sleep disorders		706 (0.5)			3884 (10.5)		
		Memory disorders		92 (0.1)			368 (1)		
		Eating disorders		71 (0.1)			79 (0.2)		
		Psychotropic drug use							
			Sedatives	4399 (2.9)			9365 (25.4)		
			Antidepressants	3320 (2.2)			15,065 (40.9)		
			Anxiolytics	7987 (5.2)			22,304 (60.6)		
			Antipsychotics	1170 (0.8)			4154 (11.3)		
	**Somatic health conditions** **, n (%)**								
		Type 2 diabetes mellitus		2983 (1.9)			1803 (4.9)		
		Hypercholesterolemia		8401 (5.5)			13,819 (37.5)		
		Metabolic dysfunction–associated steatotic liver disease		20 (0.1)			254 (0.7)		
		Hypertension		7131 (4.6)			13,343 (36.2)		
		Chronic kidney disease		398 (0.3)			653 (1.8)		
		Early heart disease		2538 (1.6)			1324 (3.6)		
		Asthma		2603 (1.7)			2393 (6.5)		
		Chronic immune inflammatory disorder		213 (0.1)			668 (1.8)		
		Cancer		1723 (1.1)			5704 (15.5)		
		Unspecified illness		1472 (1)			2986 (8.1)		

^a^All patients were followed at the Local Health Unit of Matosinhos between January 1, 2008, and May 9, 2024, and aged between 18 and 59 years.

^b^IV: interpersonal violence.

### Health Outcomes 10 Years After the Episode of Suspected Violence

**Figure 1:**
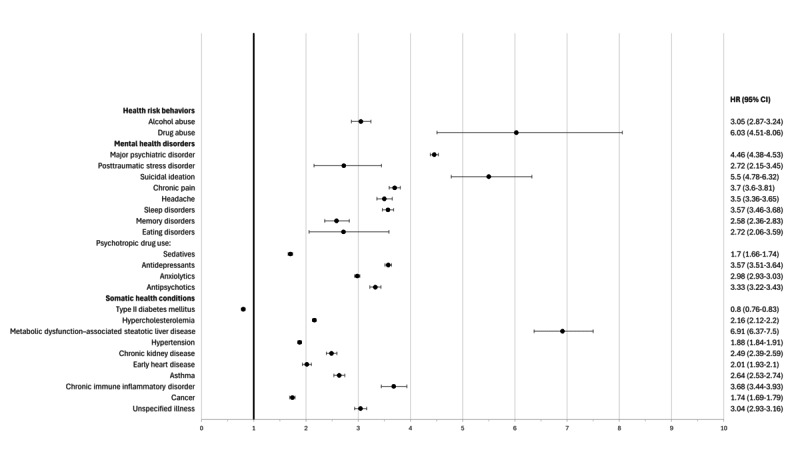
Health outcomes up to 3650 days postindex in the suspected violence cohort. All patients were followed at the Local Health Unit of Matosinhos between January 1, 2008, and May 9, 2024, and were aged between 18 and 59 years.

The results obtained from the 3650-day follow-up after the index date are represented in Figure 1 and Table 3.

For every outcome studied, the follow-up period for adult survivors of IV was consistently shorter compared to other adults. The shortest follow-up periods were observed for the use of anxiolytics (mean 1064, SD 1151), major psychiatric disorder (mean 1227, SD 1257), and hypercholesterolemia (mean 1241, SD 1171).

Health outcomes did come up earlier in survivors of IV, compared to adults who were not survivors of IV. The shortest times to event occurred in early heart disease (mean 43, SD 127), T2D (mean 123, SD 294), and hypertension (mean 381, SD 532).

The violence cohort had a higher HR of developing the outcomes studied, except for T2D (0.80, 95% CI 0.76-0.83). The outcomes that stood out, as they presented the highest HR values, were fatty liver disease (6.91, 95% CI 6.37-7.50), drug abuse (6.03, 95% CI 4.5-8.06), suicidal ideation (5.50, 95% CI 4.78-6.32), and major psychiatric disorder (4.46, 95% CI 4.38-4.53).

**Table 3:** Health outcomes after a suspected experience of IV in adults up to 10 years of follow-up^a^.

					Ev/100PY^b^			Follow-up (days), mean (SD)			Time to event, mean (SD)			HR^c^ (95% CI)
**Health risk behaviors**														
	Alcohol abuse			0.53			2244 (1285)			1055 (941)			3.05 (2.87-3.24)	
	Drug abuse			0.03			2294 (1273)			1412 (1046)			6.03 (4.05-8.06)	
**Mental health disorders**														
	Major psychiatric disorder			16.34			1227 (1257)			642 (836)			4.46 (4.38-4.53)	
	Posttraumatic stress disorder			0.04			2293 (1274)			948 (931)			2.72 (2.15-3.45)	
	Suicidal ideation			0.11			2285 (1275)			1055 (990)			5.50 (4.78-6.32)	
	Chronic pain			3.25			2007 (1291)			1295 (1014)			3.70 (3.60-3.81)	
	Headache			1.25			2177 (1283)			1378 (989)			3.50 (3.36-3.65)	
	Sleep disorders			2.75			2048 (1292)			1274 (994)			3.57 (3.46-3.68)	
	Memory disorders			0.26			2273 (1275)			1554 (1062)			2.58 (2.36-2.83)	
	Eating disorders			0.02			2294 (1273)			981 (945)			2.72 (2.06-3.59)	
	**Psychotropic drug use**													
		Sedatives	3.72			1924 (1318)			976 (970)			1.70 (1.66-1.74)		
		Antidepressants	13.36			1331 (1288)			672 (862)			3.57 (3.51-3.64)		
		Anxiolytics	20.95			1064 (1151)			646 (808)			2.98 (2.93-3.03)		
		Antipsychotics	2.56			2056 (1337)			900 (1017)			3.33 (3.22-3.43)		
**Somatic health conditions**														
	Type 2 diabetes mellitus			0.84			2174 (1328)			123 (294)			0.80 (0.76-0.83)	
	Hypercholesterolemia			16.00			1241 (1171)			808 (814)			2.16 (2.12-2.20)	
	Metabolic dysfunction–associated steatotic liver disease			0.45			2256 (1288)			996 (1092)			6.91 (6.37-7.50)	
	Hypertension			11.11			1342 (1325)			381 (532)			1.88 (1.84-1.91)	
	Chronic kidney disease			1.42			2174 (1298)			1176 (1121)			2.49 (2.39-2.59)	
	Early heart disease			0.60			2201 (1320)			43(127)			2.01 (1.93-2.10)	
	Asthma			1.20			2168 (1311)			844 (933)			2.64 (2.53-2.74)	
	Chronic immune inflammatory disorder			0.49			2245 (1288)			961 (1015)			3.68 (3.44-3.93)	
	Cancer			2.79			2026 (1324)			915 (997)			1.74 (1.69-1.79)	
	Unspecified illness			1.59			2133 (1291)			1242 (1003)			3.04 (2.93-3.16)	

^a^All patients were followed at the Local Health Unit of Matosinhos between January 1, 2008, and May 9, 2024, and aged between 18 and 59 years.

^b^Ev/100PY: event rate per person-year.

^c^HR: hazard ratio.

## Discussion

### Principal Findings

The analysis of EHR data from adult users of ULSM revealed that patients who were suspected survivors of IV were more likely to develop negative outcomes when compared to the control cohort. This was true for the different health outcomes regarding health risk behaviors, mental health, and somatic conditions studied, except for T2D. In addition to reinforcing existing literature on the subject, we advanced a potential link between the experience of violence and the onset of MASLD.

We found that adult suspected survivors of IV were more likely to develop drug and alcohol abuse, respectively. This goes according to Rivara et al [6], Stubbs and Szoeke [12], Kyle [20], and Colaprico et al [21], which describe a relation between different types of IV and alcohol and substance use. Health risk behaviors, as substance abuse and alcohol abuse, may work as coping mechanisms to violence exposure [6,20,21], but it is important to note that this relationship is bidirectional. Substance abuse and worsened mental health may result in a higher risk of being a survivor of violence or inflicting harm on others [20].

According to WHO, “a mental disorder is characterized by a clinically significant disturbance in an individual’s cognition, emotional regulation, or behavior” [22]. In this study, adult suspected survivors of IV were more likely to develop each of the mental disorders studied. This goes according to other studies, such as Rivara et al [6], which summarizes the relation between intimate partner violence and depression, posttraumatic stress disorder, suicidal ideation, and chronic mental illness. Kyle [20] and Stubbs and Szoeke [12] describe similar findings, adding anxiety disorders and eating disorders, respectively. In this study, suspected survivors of IV were more likely to develop memory disorders, which is consistent with findings described by Campbell et al [23].

Sexual abuse is also related to depression, anxiety, sleep disorders, suicide, eating disorders, and posttraumatic stress disorders, according to Rivara et al [6]. Bullying and cyberbullying in the youth and children population seem to be linked to depression, anxiety, self-esteem, posttraumatic stress disorder, sleep disturbances, and headache [6]. Colaprico et al [21] described a relation between workplace bullying in adults and disorders as anxiety, depression, suicidal tendencies, and headache.

Another finding of this study was the higher likelihood of the use of psychotropic drugs. This finding seems to be directly related to the type of mental health disorders developed and their comorbidities, as these drugs may be part of a treatment strategy to respond to them.

Literature describes correlations between IV in adults and various health issues, including cardiovascular diseases, hypertension, elevated cortisol levels, cancer, sexually transmitted infections, a higher risk of developing cancer, gastrointestinal disorders, such as irritable bowel syndrome, and asthma [5-7,12].

Lin et al [24] found that children exposed to adverse childhood experiences had higher risks of dyslipidemia, asthma, liver disease, kidney disease, and arthritis, similar to our findings in the adult population.

We found a potential link between adult IV and MASLD. This possible correlation with violence has not yet been described in the literature for the adult population. However, a recent cross-sectional study found a moderate risk in men for specific forms of child maltreatment [25]. Recent studies have focused on the relation between NALFD and mental health disorders. Shea et al [26] described in their systematic review a high prevalence of depression, anxiety, and stress among adults with MASLD. Kang et al [27] concluded that higher perceived stress was independently associated with an increased prevalence of MASLD. Soto-Angona et al [28] have also come to the conclusion that MASLD is part of a complex system of mental and noncommunicable somatic disorders, with a shared underlying pathogenesis. This is based on common lifestyle and environmental risk factors, mediated by dysregulation of inflammation, oxidative stress pathways, and mitochondrial function.

This study found an inverse correlation between the experience of IV and T2D. Some studies have found a link between intimate partner violence and T2D [7,10,12]. Studies performed on adverse childhood experiences also correlate these experiences to the higher prevalence of T2D in childhood [29,30] This way, the lower HR of developing T2D in the IV cohort may be explained by a higher prevalence of the disease in children exposed to violence—a higher baseline prevalence could lead to lower incidence in adulthood as a bigger percentage of the cohort could have developed the disease prior to index date [30]. In this study, 4.9% of the IV cohort had T2D at the index date compared to only 1.9% of the control cohort.

Survivors of violence may be more susceptible to losing follow-up due to instability in their lives, which can result in missed appointments, relocation, or increased mortality, for example.

As previously said, the association between IV and negative health outcomes may be partially explained by the impact of chronic stress on various systems that regulate body homeostasis, particularly the HPA axis [5,8,16,31,32]. Hyperactivation of the HPA axis leads to neuroendocrine and immune disruption and altered systemic cortisol production [5,8,31]. Survivors of violence are at a higher risk of resurvivorization across their lifespan, exposing them to chronic and cumulative stress. This snowball effect may result in the negative outcomes explored in this study [16,32]. In fact, violence may even start before birth (intrauterine violence), in cases of violence against pregnant women, perpetuating through every phase of life, having a cumulative effect with tremendous consequences [16].

Even though it seems clear that violence is deleterious, we are still not able to comprehend the full extent of its consequences. Studies tend to address IV within each subtype of violence or a specific subgroup of the population, overlooking the intricate relation between different forms of violence and recurrent experiences of it. This limits our capacity to draw conclusions and compare patterns across diverse populations. A lot of studies have been done on children, trying to understand the direct impact of violence on health during childhood, but also its long-term consequences through their adult life [4,8,29-31,33-35]. There is also a great amount of literature regarding intimate partner violence, particularly among women [7,12,15,20,23,36-38]. However, there is limited research on other subtypes of IV. The body of research regarding men’s health and violence is less robust than that for women [18], creating barriers in understanding the true burden in this sex and whether there are significant differences in response to violence between sexes.

Since multiple experiences of violence across a person’s lifespan are common, we will only understand the full impact of IV by considering the co-occurrence and interconnections of the multiple experiences of it. Violence must be conceptualized from a dose-response perspective, considering the accumulation of exposure over time and across multiple domains [19]. Scott-Storey et al [32] propose an approach aligned with this. They developed a cumulative lifetime violence severity scale reflecting dimensions of type, focus (target or perpetrator), timing (childhood or adulthood), context, frequency, and degree of distress. This tool was designed only for men. Similar tools could be developed for both sexes, adjusting to each population, allowing a profound understanding of violence’s negative impact on health.

In this study, we chose to have a global approach to IV in adults, as we could not develop a dose-response perspective or differentiate each type of violence in each patient, due to the poor codification on this matter and the broad vocabulary used to describe it in EHR.

Our results underscore the significant burden of IV, which affects multiple facets of survivors’ lives. Health consequences have their own repercussions that substantially diminish survivors’ quality of life, affecting other spheres of life, such as job loss and career abandonment, or increased health care costs [1,15]. Violence is therefore conceptualized as a structural determinant of health. Its presence negatively impacts patients’ health outcomes and self-development. Moreover, distinct patterns of cumulative lifetime exposure to violence result in varying degrees of symptom severity [13].

Violence screening represents a cost-effective prevention strategy that should be standard practice [39]. Several barriers prevent an effective detection of the cases: (1) survivors may struggle with disclosure due to shame, fear of being judged, of not being believed [40], or of violence escalation; (2) health care practitioners may face challenges such as insufficient time and skills to identify cases, discomfort, confidentiality concerns, or their own experiences with IV [39,40]; and (3) clinical-level factors may include inadequate information, lack of screening protocols, and insufficient support [39,40]. These constraints may lead to a misdiagnosis of cases. Furthermore, due to medical privacy and doubts associated with the case complexity, some situations might only be recorded in confidential registers, which may contribute to an apparent underidentification of IV cases, making it difficult to understand the full extent of the problem.

Since almost all clinicians across their practice will encounter patients who were survivors of IV, they should be trained with the necessary skills to identify and report these situations and respond appropriately, reducing their harmful consequences and preventing reoccurrence [37,39,40]. Public institutions must have a direct role in the prevention of violence, addressing underlying causes, and creating appropriate measures and programs that can give the needed support to survivors and their families.

A deeper understanding of the long-term consequences of IV enables an improvement in the approach to survivors in medical practice. By identifying the negative health outcomes that may arise from exposure to violence, clinicians can develop a proactive and targeted follow-up of survivors. An early detection of these pathologies and an understanding of the complex and multifaceted needs of survivors contribute to a more personalized, preventive, and effective medical care.

### Limitations

Omission bias is possible due to certain aspects of this study. Physicians might not document or code all suspected IV cases they detect in the EHR. Additionally, they may choose to record these cases only in confidential health registers.

Given that the identification of suspected cases of IV was done through data from the EHR of adult users, we implemented comprehensive selection criteria and used a dual approach that integrates all relevant ICPC-2, *ICD-9*, and *ICD-10* codes, as well as all relevant clinical notes at every intervention point for any health care professional.

While we tried to include pertinent and broad terminology used by physicians to describe violence scenarios in clinical notes, it is not possible to account for the all variety of possible expressions that may have been used to describe a context of violence; as such, there may be a potential subestimation of violence-exposed patients in the respective cohort.

Furthermore, the variability in coding and terminology used to describe violence between countries and institutions may compromise the comparability and reproducibility of our findings when applied to different clinical settings.

Another potential limitation of this study is the reporting bias that may be associated with patients who are suspected of IV, as health care professionals may have a stronger tendency to report health care outcomes, especially those connected with mental health, regarding these patients.

In addition, given the fact that there is no methodical registration of variables as socioeconomic status, race or ethnicity, and educational level, it was not possible to adjust the data for these possible confounders; therefore, confounding bias may be present.

By including all the patients who met the inclusion criteria, without any additional exclusion criteria, we minimized selection bias and enhanced the detection of patients exposed to violence. Our comprehensive approach, encompassing all the eligible population at ULSM during the study period, resulted in a large and diverse sample, which strengthens the validity and generalizability of our findings.

A validation study of these data should be carried out. We intend to extend the study to other regions of Portugal and collaborate with research groups in other countries, which would allow us to assemble larger cohorts, conduct robust statistical analyses, and gain a deeper understanding of violence to improve prevention, intervention, and treatment. The use of real-world data is recommended as it provides the possibility of analyzing larger patient populations over longer timelines [41]. We also intend to compare the effects of IV between sexes and to develop study designs that incorporate the cumulative impact of violence across multiple domains and stages of life, while characterizing the studied populations with further socioeconomic demographic data such as profession, employment situation, level of education, and family structure.

### Conclusions

This research has demonstrated that adults suspected to be survivors of IV are more likely to develop negative health outcomes than the general population. This was true for health risk behaviors, as well as for mental health and somatic conditions. Notably, this study introduces a novel perspective on the matter, suggesting that violence is linked with the development of MASLD.

Furthermore, adults suspected to be survivors of IV developed health outcomes earlier and had a shorter period of follow-up, compared to the adults with no exposure to violence. These findings highlight the complex impact of violence on health, underscoring the urgent need to address this endemic problem in society. Health providers play a crucial role in the early detection of cases, follow-up, and treatment of survivors of IV.

Future multicentric research (including international research groups) should focus on validating these findings, trying to have a dose-response perspective. Such efforts will enable more robust statistical analyses and a deeper understanding of violence’s consequences, ultimately contributing to improved prevention, detection, and treatment strategies.

## Abbreviations

EHR: electronic health record
HPA: hypothalamic-pituitary-adrenal
HR: hazard ratios
ICD-9: International Classification of Diseases, Ninth Revision
ICD-10: International Statistical Classification of Diseases, Tenth Revision
ICPC-2: International Classification of Primary Care, 2nd edition
IV: interpersonal violence
MASLD: metabolic dysfunction–associated steatotic liver disease
T2D: type 2 diabetes mellitus
ULSM: Unidade Local de Saúde de Matosinhos
WHO: World Health Organization

## Data Availability

Data sharing is not applicable to this article as no datasets were generated during this study. Patient-level data used in this study are not publicly available, as the Zero Trust model was used. Researchers accessed only aggregated and anonymized results, ensuring full compliance with data protection, ethical, and privacy standards. This article incorporates all the available statistical results.
